# Rescue of cognitive and negative-like symptoms by chronic aripiprazole treatment in a 3-hit mouse model of neurodevelopmental disorder

**DOI:** 10.3389/fphar.2026.1760743

**Published:** 2026-03-16

**Authors:** Imane Mouffok, Noa Roudaut, Cécile Chofflet, Thomas Freret, Michel Boulouard, Valentine Bouet

**Affiliations:** University of Caen Normandy (UNICAEN), Institut National de la Santé et de la Recherche Médicale (INSERM), Mobilités : Vieillissement, Pathologie, Santé (COMETE), CYCERON, Centre Hospitalier Universitaire (CHU) Caen, Normandie Université, Caen, France

**Keywords:** coat grooming, glutamate, multifactorial model, pharmacological models of schizophrenia, phencyclidine, recognition memory, selfcare, working memory

## Abstract

**Introduction:**

Cognitive and negative symptoms remain among the most disabling features of schizophrenia but are still poorly addressed by existing antipsychotics. Advances in therapeutic development are hampered by the lack of preclinical models that adequately reflect the disorder’s multifactorial etiology, and by their limited ability to encompass the broad and heterogeneous symptom spectrum. The development of refined, translationally relevant models is therefore critical to advance both mechanistic understanding and therapeutic innovation.

**Methods:**

Here, we assessed in male and female mice the characterization of an innovative 3-hit mouse model that integrates genetic, environmental, and pharmacological risk factors, with a particular emphasis on the glutamatergic deficit hypothesis. The model combines: serine racemase deletion (reduced NMDA receptor co-agonist synthesis), maternal separation at postnatal day 9 (early-life stress), and subchronic phencyclidine exposure (NMDA receptor antagonism). Moreover, to assess the predictive validity of the model, a group of 3-hit mice was treated with chronic aripiprazole (1 mg/kg).

**Results:**

Behavioral analysis showed that 3-hit mice display a broad schizophrenia-like phenotype, marked by hyperlocomotion, memory deficits, impaired social recognition, social withdrawal, and apathy-like behavior. Importantly, chronic aripiprazole attenuated most of these alterations, underlying the predictive validity of the model.

**Discussion:**

By integrating multiple causal dimensions, this 3-hit combination provides a relevant way to induce a complex phenotype with schizophrenia-like behavioral keys, offering a new preclinical model to explore therapeutic strategies for schizophrenia profiles with cognitive and negative deficits.

## Graphical Abstract

**Figure:**
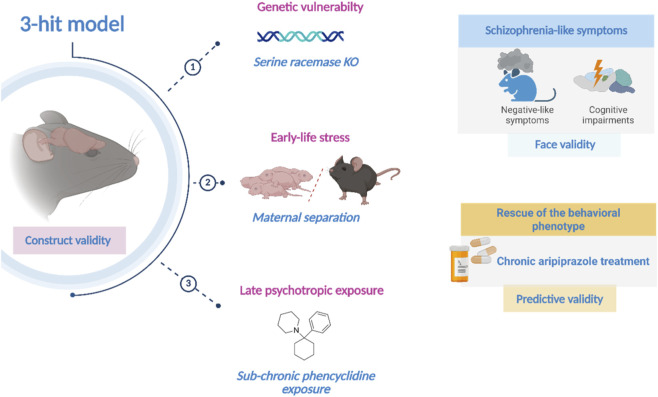

## 1 Introduction

Schizophrenia is a severe and life-long psychiatric disorder that starts during adolescence and affects about 1% of the population (Solmi et al., 2023). The disease is described by the presence of three categories of symptoms: positive symptoms (hallucinations, delusions, etc.), negative symptoms (anhedonia, social withdrawal, lack of motivation, etc.), and cognitive impairments (attention, working memory, executive functions) (Faden and Citrome, 2023; Gebreegziabhere et al., 2022; Marder and Umbricht, 2023). If schizophrenia affects both sexes, notable differences have been documented, particularly in age at onset, symptoms severity, disease course, and overall prognosis. Taken together, these factors tend to result in a more favorable clinical trajectory in women (Gogos et al., 2019; Gogos et al., 2015). Current antipsychotic treatments, which primarily act on dopamine D_2_ receptors, are generally effective in reducing positive symptoms. Yet, up to 30% of patients show only limited or no improvement and are considered to be treatment-resistant. In addition, these drugs display limited efficacy for negative and cognitive symptoms, the dimensions that most strongly drive disability and poor long-term outcomes (Stępnicki et al., 2018). These observations suggests that therapeutic approaches based primarily on modulating dopamine receptors cannot address the full range of symptoms associated with schizophrenia.

Over the past two decades, new animal models combining genetic factor, early and late environmental factors named combined models have emerged (Ellenbroek, 2003; Percelay et al., 2024). In this context, and considering the hypothesis of NMDA hypofunction, we have recently described behavioral phenotype of a new 3-hit model in mice (Mouffok et al., 2024). The first hit is the partial deletion of the *serine racemase* (SRR) gene, which reduces forebrain D-serine levels by ∼90% (Ploux et al., 2020). D-serine is the main endogenous co-agonist at the glycine modulatory site of NMDA receptors. Polymorphisms in *SRR* and reduced brain D-serine levels have been reported in patients with schizophrenia; linking this pathway to impaired NMDA-R function (Balu and Coyle, 2015; Coyle, 2024). The second hit is maternal separation (MS) during early postnatal life, a well-established paradigm for early environmental stress. In rodents, MS has been demonstrated to activate hypothalamus-pituitary axis (van Bodegom et al., 2017), to alter NMDA receptor expression (Wilber et al., 2009), and to impair social behavior (Bouet et al., 2011). This manipulation models an early-life environmental risk factor consistent with the neurodevelopmental origins of schizophrenia (Meyer and Feldon, 2010; Murray and Lewis, 1987; Weinberger, 1987). The third hit is subchronic (14 days) phencyclidine (PCP, non-competitive NMDA antagonist) exposure in adulthood, a pharmacological challenge that affects NMDA-R function. Repeated PCP administration rapidly induces both positive and negative mimicking symptoms of schizophrenia as well as stable cognitive impairment in rodents (Li et al., 2024). We previously demonstrated that this model induced in females the triad of schizophrenia-like symptoms and is particularly characterized by robust (persistent) alterations in social behavior (Mouffok et al., 2024).

The first objective of the study presented herein was to assess the predictive validity of the model. For this purpose, we used aripiprazole (ARZ), a third-generation antipsychotic, with a partial agonist activity on dopamine D2, D3 receptors, serotonin 5-HT1A receptor, as well as an antagonist activity on serotonin 5-HT2A receptor (Preda and Shapiro, 2020). Its pharmacological properties regulate positive schizophrenic symptoms and, to a lesser extent, negative symptoms and cognitive deficits, with minimal metabolic and extrapyramidal side effects (Hori et al., 2017). In particular, ARZ demonstrated effectiveness in treating both positive and negative symptoms in the context of acute intervention, or for long-term maintenance (Huhn et al., 2019; Kane et al., 2002; Potkin et al., 2003; Schneider-Thoma et al., 2022). Currently, ARZ is approved for use in patients with various psychotic disorders, including schizophrenia, bipolar disorder, depression, Tourette’s Disorder, and autism (Kirino, 2012; Pae, 2009; Pae et al., 2011). Thus, among atypical antipsychotics, ARZ can be considered as a clinical reference (widely prescribed), and as a pharmacological reference (first partial dopamine receptor agonist approved). From a practical standpoint, and given the long, chronic administration of the ARZ treatment in the study presented here (9 weeks), we chose a recently validated administration method using a solution dispensed in drinking water (Chofflet et al., 2025). Indeed, even with extensive training of the experimenter and as habituation of the animals, repeated oral gavage can cause a significant level of animal discomfort and stress, posing a major limitation in psychiatric research and in animal welfare. The second objective of the study was to investigate more deeply the face validity of this model, in both male and female mice, with a particular focus on negative-like symptoms and cognitive deficits. To this, spontaneous behavior, working memory, sociability, object and social recognition, self-care and nesting behaviors were assessed.

## 2 Materials and methods

### 2.1 Animals

Animal used were wild-type male and female C57BL/6J mice and serine racemase knockout (SRKO) mice (Basu et al., 2008), originally derived from Dr. Coyle’s laboratory colony (Belmont, MA, United States), and maintained at the local animal facility (Centre Universitaire de Ressources Biologiques, University of Caen Normandy, Caen, France). They were housed in standard laboratory cages (4-7 animals per cage) under controlled environmental conditions: reversed 12 h light/dark cycle (lights on at 7:30 p.m.), ambient temperature of 21 ° ± 1 °C, and relative humidity of 55% ± 10%. Animals had free access to food and water and were weekly weighted. Enrichment consisted in crinkle-cut paper and cardboard shelters. Post-weaning identification was achieved by subcutaneous implantation of electronic microchips under isoflurane anesthesia (5% for induction, 2.5% for maintenance) in an oxygen/nitrous oxide mixture (30%/70%). Behavioral testing was conducted between 11 and 15 weeks of age. All procedures were performed in accordance with French and European guidelines for the ethical use of laboratory animals (Directive 2010/63/EU), approved by the French ministry of research under the number APAFIS#49215-2024042615112665-v3.

### 2.2 Maternal separation (MS)

To model early-life stress, maternal separation was conducted on postnatal day 9 (PND9). Half of the wild-type and SRKO litters were subjected to a 24 h maternal separation protocol (Bouet et al., 2011; Ellenbroek, 2003; Mouffok et al., 2024). Dams were temporarily relocated into individual cages placed near their respective litters at 9:00 a.m. After 24 h (or 20 s for control), dams were returned to their pups and left undisturbed until weaning.

### 2.3 Pharmacological treatment

#### 2.3.1 PCP administration

At 8 weeks of age, mice received daily subcutaneous (s.c.) injections of freshly dissolved phencyclidine hydrochloride (PCP; Sigma-Aldrich, Saint-Quentin-Fallavier, France) in saline at the dose of 10 mg/kg, in a volume of 10 mL/kg, for 14 consecutive days (Mouffok et al., 2024; Noda et al., 1995). Control animals received equivalent volumes of saline. A 1-week washout followed the treatment phase prior to the initiation of ARZ administration.

#### 2.3.2 Aripiprazole administration

Aripiprazole hydrochloride (Thermo Fisher Scientific©, Waltham, United States) was dissolved in water containing 0.5% polysorbate 20 (Tween 20®, Sigma-Aldrich©, Saint-Louis, United States) and acidified with two drops of glacial acetic acid per 100 mL, as we previously described (Chofflet et al., 2025). The ARZ solution concentration was adjusted every 3 days for each cage according on the hydric consumption of mice, in order to deliver a target dose of 1 mg/kg/day. The treatment was administered for a total duration of 6 weeks, starting 1 week after the last PCP administration which corresponds to 3 weeks prior to the onset of behavioral testing. It was maintained continuously throughout the entire behavioral assessment period.

### 2.4 Behavioral assessments

#### 2.4.1 General procedure

**FIGURE 1:**
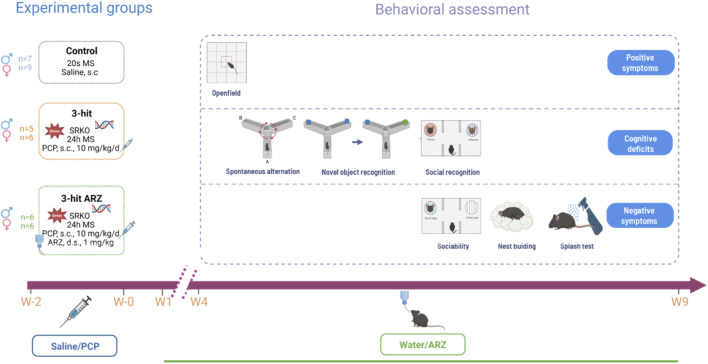
Experimental groups and design of the behavioral assessment. The 3-hit group contained serine racemase KO (SRKO), maternally separated (MS for 24 h at the age of 9 days) and phencyclidine treated mice (PCP, 10 mg/kg, s.c, daily for 2 weeks). The 3-hit ARZ mice received aripiprazole (1 mg/kg/day) via drinking solution from 3 weeks prior to the onset and throughout the entire behavioral testing period, lasting from the 4th to the 9th weeks after the end of PCP/Saline treatment. s.c: subcutaneous; d.s: drinking solution W: Week. ARZ: aripiprazole.

All animals were daily handled for 5 min during the week preceding behavioral testing to familiarize them to humans and reduce stress. Testing took place during the active phase of the reversed light/dark cycle, beginning at 9:00 a.m. Animals were acclimated to the experimental room for 30 min before each behavioral testing. The experimental design included three groups: control, 3-hit and 3-hit chronically treated with ARZ (Figure 1). Behavioral assessments were carried out between the 4th and the 9th weeks after the last PCP (or saline) administration, using a comprehensive battery of tests targeting schizophrenia-like dimensions, including positive-like symptoms, negative-like symptoms, and cognitive deficits. ARZ treatment continued during all the behavioral assessment. Each behavioral test was recorded and analyzed with EthoVision XT v18.0 software (Noldus®, Wageningen, Netherlands). The experimenter was blind to the experimental groups.

#### 2.4.2 Open field test

To assess general locomotor activity, exploratory behavior, and anxiety-related responses, mice were individually placed in a square white open field arena (50 cm × 50 cm × 50 cm) under low illumination (15 lux) for 10 min (Mouffok et al., 2024; Prut and Belzung, 2003). Locomotor activity (total distance traveled, number of rearings) as well as time spent in the central zone of the arena were extracted from video tracking as indices of activity and anxiety-related behavior, respectively. As for all experiments, the apparatus was cleaned with 70% ethanol between animals to remove scent cues.

#### 2.4.3 Spontaneous alternation test

Spatial working memory was assessed in the Y-maze spontaneous alternation task, following previously described protocols (Mouffok et al., 2024). The maze consisted of three identical black-painted wooden arms (22 cm × 6.5 cm × 10 cm) arranged at 120° angles and illuminated at 20 lux. Each mouse was placed in the center of the maze and allowed to freely explore for 10 min. An arm entry was defined as all four paws entering an arm. The sequence of entries was used to calculate the percentage of spontaneous alternation using the formula: Number of 3-arm alternations/(Total number of entries −2) × 100. This value was compared to 50%, which indicates a random exploration of the maze (chance) and impaired working memory.

#### 2.4.4 Object recognition test

The object recognition protocol was carried out in the same Y-maze apparatus in two phases (Darwish and Hasan, 2019; Leger et al., 2013). The previous spontaneous alternation session was used as a habituation session to the maze. The day after, for the acquisition phase, each mouse was placed in the maze for 10 min, where two identical objects (either Lego® towers or Falcon® flasks filled with sand, randomized) were positioned at the ends of two arms. Then, the mouse was returned to its home cage for an intertrial interval of 10 min. For the retention phase, one of the objects was replaced by a novel object, and the mouse was again allowed to explore for 10 min. The positions of the familiar and the novel object were randomized between arms and across animals to avoid potential side preference. Object exploration was manually scored and defined as the mouse orienting its nose towards the object within 1 cm, sniffing or touching it. Climbing or sitting on the object was not considered as exploration. Objects were cleaned with 70% ethanol between trials to remove scent cues. A discrimination index (DI) was calculated as follows: (Exploration time of the novel object – Exploration time of the familiar object)/Total exploration time (de Bruin and Pouzet, 2006).

#### 2.4.5 Sociability and social recognition test

Social behavior was assessed with the three-chamber social interaction paradigm, as previously described (Lahogue et al., 2025; Mouffok et al., 2024; Moy et al., 2004). The apparatus consisted of a transparent Plexiglas box (60 cm × 45 cm × 22 cm) divided into three equally sized chambers (20 × 45 × 22 cm), connected by small openings to allow free exploration of the three compartments. In each of the two side chambers, a cylindrical wire cage (8.5 cm in diameter × 16.5 cm in height) was placed to house a stimulus mouse or to remain empty. The test was conducted under dim lighting (20 lux) and began by a 5-min habituation phase, during which the subject mouse was allowed to explore all three chambers freely, with both wire cages empty. Immediately after habituation, a novel, juvenile sex-matched C57BL/6J mouse (4 weeks old), with no prior interaction with the subject, was placed inside one of the wire cages. The position of the stimulus mouse (left or right chamber) was alternated across animals to avoid potential side preference. During this phase of 10-min, social interaction behaviors towards the stimulus mouse were collected: direct nose contact, sniffing (less than 1 cm), and rearing toward the wire cage. Social preference was thereafter assessed by measuring the total exploration time of each cage. After a 5-min delay, a social recognition test was conducted to assess the subject’s preference for social novelty. In this phase, the previously encountered stimulus mouse (familiar) remained in the wire cage, while a second, novel mouse (juvenile and sex-matched) was introduced into the previously empty cage. During a new 10-min phase, the subject’s interaction time with each stimulus mouse (familiar vs. novel) was recorded using the same criteria. A sociability index (SI) and a social discrimination index (SDI) were calculated respectfully as follows: SI = (Interaction time with the stimulus mouse – Interaction time with the empty wire cage)/Total exploration time; SDI = (Interaction time with the unfamiliar mouse – Interaction time with the familiar mouse)/Total exploration time. Locomotor activity (distance traveled) and its space distribution as well as time spent in each compartment were collected thanks to videotracking.

#### 2.4.6 Splash test

The splash test was employed to evaluate self-care and goal-directed behavior, serving as an index of apathy-like symptoms (Okitsu et al., 2024). Prior to testing, each mouse underwent a 5-min habituation phase in a clean, empty standard cage containing bedding and maintained under low-lighting (30 lux). Immediately afterward, the mouse back was sprayed with a 10% sucrose solution to induce grooming (Deore et al., 2024; Ducottet and Belzung, 2004). Behavior was recorded over a 5-min period, and two parameters were measured: the latency to initiate grooming and the total duration of grooming. Increased latency and reduced duration of grooming are indicators of diminished motivational drive (Becker et al., 2021).

#### 2.4.7 Nesting behavior

Nesting has been shown to be disrupted in models of neuropsychiatric disorders, in particular in schizophrenia models (Kim et al., 2022; Pedersen et al., 2014; Sandhu et al., 2014). Mice were individually housed for 24 h in standard cages containing bedding and cardboard house but no other enrichment items. One hour prior to the onset of the dark cycle, a single pressed cotton Nestlet (5 cm × 5 cm; Serlab©, Montataire, France) was placed in the center of the cage. Twenty hours later, nest construction was scored by an experimenter blinded to experimental conditions using a 5-point score (Deacon, 2006). 0: Nestlet untouched (>90% intact); 1: Nestlet partially shredded (50%–90% intact), no defined nest site; 2: Nestlet mostly shredded, material spread but no structure; 3: Partial nest with walls, not exceeding body height; 4: Well-formed nest with walls near body height; 5: Fully enclosed, dome-like nest with a defined cavity.

### 2.5 Statistical analysis

All statistical analyses were primarily conducted using GraphPad Prism version 8.3 (GraphPad® Software, San Diego, CA, United States), with supplementary analyses performed with RStudio version 3.5.0 (R Foundation for Statistical Computing, Vienna, Austria). The normality of data distributions was assessed using the Shapiro–Wilk test, and homogeneity of variances between groups with Levene’s test. When data distributions met parametric assumptions, group differences were analyzed using one-way or two-way analysis of variance (ANOVA), followed by appropriate *post hoc* multiple comparison procedures, such as Tukey’s or Bonferroni’s tests. For data that were not normally distributed, non-parametric methods were employed, notably the Kruskal–Wallis test. In the spontaneous alternation test, performances were compared to the theoretical chance level of 50% using a one-sample t-test. Similarly, in the novel object recognition, sociability, and social recognition tasks, discrimination indices were compared to the theoretical baseline of 0 using one-sample t-tests. For the novel object recognition and social recognition tasks, data were analyzed using two-way repeated-measures ANOVA to compare responses to different stimuli across groups.

All data are reported as mean ± standard error of the mean (SEM) for normally distributed variables, or as median ± interquartile range for non-normally distributed data. A p-value lower than 0.05 was considered statistically significant.

## 3 Results

### 3.1 Open field test

**FIGURE 2:**
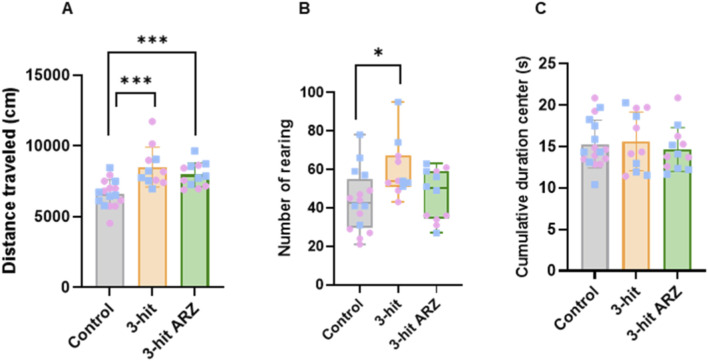
Spontaneous locomotor activity and anxiety-like behavior in open field in 3-hit and 3-hit mice treated with aripiprazole (3-hit ARZ). **(A)** Distance traveled (cm). **(B)** Number of rearing. **(C)** Cumulative duration (s) in the center of the arena. Data are presented as mean ± SEM or as median ± IQT. Intergroup comparisons were conducted using either one-way ANOVA or Kruskal–Wallis tests, followed by Tukey’s or Dunn’s *post hoc* tests; *p < 0.05; ***p < 0.001. Females are indicated in pink circles and males in blue squares. Control n = 16; 3-hit n = 11; 3-hit ARZ n = 12.

Distance traveled in the open field globally differed between groups (one way ANOVA, F = 11,04; p = 0,0002; Figure 2). Post-hoc analyses showed that both 3-hit and 3-hit ARZ groups of mice displayed a significant increase in horizontal activity relative to controls (respectively, Tukey’s post-hoc p = 0.0002 and 0.0073). Similarly, a group difference was observed for vertical activity (Kruskal-Wallis, p = 0,0366). Post-hoc analyses revealed that only the 3-hit mice group displayed a higher number of rearing than control mice (Dunn’s post-hoc p = 0.0322). In addition to horizontal and vertical activity, the time spent in the central zone of the open field was collected but did not reveal significant differences between groups.

### 3.2 Memory tests

**FIGURE 3:**
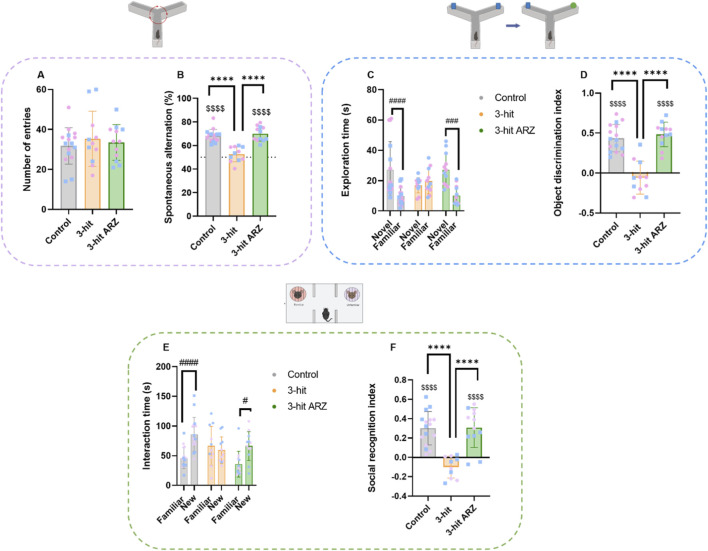
Working memory, object and social recognition performances in 3-hit and aripiprazole (ARZ) 3-hit treated mice. **(A,B)** Spontaneous alternation in Y-maze: numbers of arm entries **(A)** and alternation percentage **(B)**. **(C,D)** Novel object recognition: exploration time of the novel and familiar objects **(C)** and object discrimination index **(D)**. **(E,F)** Social recognition: exploration time of familiar and novel mice **(E)** and social recognition index **(F)**. Intergroup comparisons were conducted using either one-way ANOVA or Kruskal–Wallis tests, followed by Tukey’s or Dunn’s *post hoc* tests: ****p < 0.0001; comparisons with a reference value (50% for spontaneous alternation % and 0 for both indices) were conducted using a one sample t-test: $$$$ p < 0.0001; comparison between novel and familiar objects or mice were conducted using two-way ANOVA: #p < 0.05 ###p < 0.001; ####p < 0.0001. Control n = 16; 3-hit n = 11; 3-hit ARZ n = 12.

Regarding working memory evaluation in the Y-maze, it worth to note that no group difference was observed in the total number of arm entries (one way ANOVA, F = 0.3828, p = 0.6847) (Figure 3A), indicating a similar level of exploratory behavior. Besides, while control animals exhibited alternation percentage significantly above the chance level of 50% (one sample t-test p < 0.0001), this was not the case for 3-hit mice. Such result argues for a significant impairments of working memory performances (Figure 3B). Remarkably, treatment with ARZ led to a restoration of spontaneous alternation performances, with 3-hit ARZ mice having an alternation percentage statistically above 50% (one sample t-test p < 0.0001). Besides, group comparisons revealed a difference (one-way ANOVA, F = 30,25, p < 0.0001). Post-hoc analyses revealed significant differences in alternation percentage between 3-hit and 3-hit ARZ groups (Tukey’s test p < 0.0001).

Regarding the novel object recognition test, total exploratory time during both the acquisition and retention phases did not differ between groups (Kruskal–Wallis test, data not shown). In addition, two-way repeated-measures ANOVA analysis revealed a significant object effect and a group × object interaction (respectively, F = 31.33 p < 0.0001, and F = 11.61 p = 0.0001), but no group effect (Figures 3C,D). Discrimination index, as a contrary, differed between groups (one-way ANOVA, F = 33,58, p < 0.0001). Control animals displayed a significantly longer exploration time of the novel object than of the familiar one (Bonferroni’s post-hoc p < 0.0001) with a high discrimination index significantly above chance level (one sample t-test p < 0.0001) showing a clear preference for the novel object. In contrast, 3-hit animals explored the novel and familiar objects for comparable durations, as reflected by a significantly lower discrimination index compared to controls (Tukey’s post-hoc, *p* < 0.0001). Moreover, their discrimination index did not differ from 0, which corresponds to a no-discrimination score. The 3-hit ARZ mice spent significantly more time exploring the novel object compared to the familiar one (Bonferroni’s post-hoc p < 0.0001), and displayed a discrimination index significantly higher than that of the untreated 3-hit group controls (Tukey’s post-hoc p < 0.0001). The discrimination index of the 3-hit ARZ group was also significantly above 0 (one sample t-test p < 0.001). These results indicate that ARZ treatment successfully restored recognition memory performance in the 3-hit model.

As regards to social recognition performances (Figures 3E,F), two-way repeated-measures ANOVA analysis showed a significant stimulus effect and a group × stimulus interaction (respectively, F = 13.73 p = 0.0004 and F = 6.177 p = 0.0033), but no group effect. In addition, social recognition index differed between groups (one-way ANOVA, F = 33,58, p < 0.0001). As expected, control animals showed a significant preference to more time investigating the novel mouse than the previously encountered one (Bonferroni’s post-hoc p < 0.0001). This was confirmed by a social recognition index significantly above 0 (one sample t-test p < 0.0001), indicating a clear preference for the novel mouse. On the other hand, 3-hit mice failed to show any preference, investigating the familiar and the novel mice with similar amount of time. Their social discrimination index was consequently not statistically different from 0, reflecting a deficit in social recognition memory. Importantly, 3-hit ARZ mice spent significantly more time exploring the novel conspecific compared to the familiar one (Bonferroni’s post-hoc p < 0.0105), and their discrimination index was significantly higher than the 3-hit group (Tukey’s post-hoc p < 0.0001) and statistically above chance level (one sample t-test p < 0.0001). These findings indicate that ARZ treatment effectively rescues social recognition memory performances in the 3-hit model.

### 3.3 Sociability and apathy tests

**FIGURE 4:**
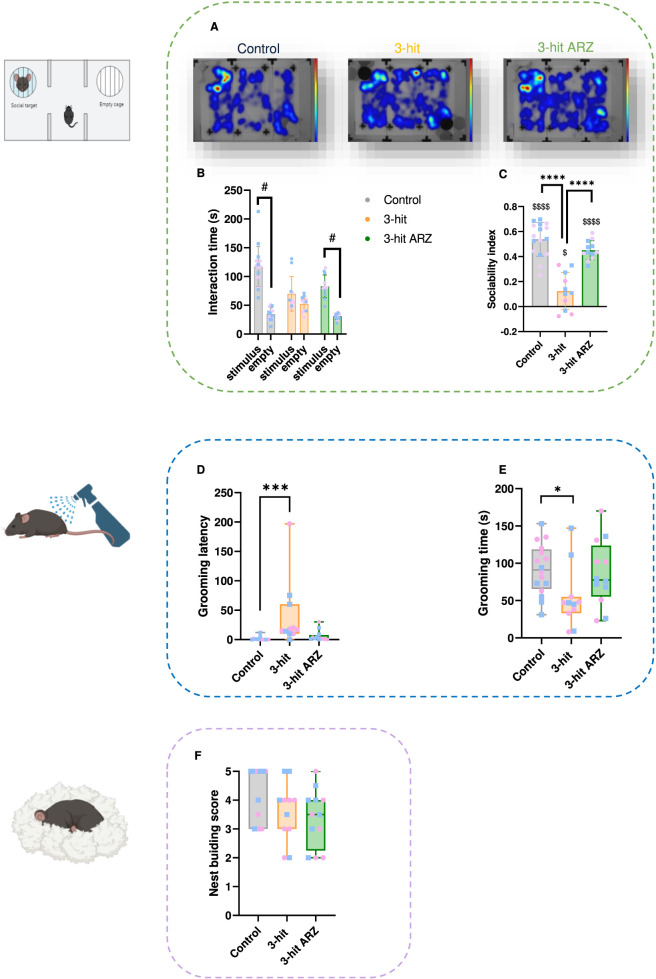
Social and selfcare behavior in 3-hit and aripiprazole (ARZ) 3-hit treated mice. **(A–C)** Social behavior in the three-chamber sociability test. **(A)** Interaction time with the cage containing the stimulus mouse and the empty cage. **(B)** Sociability index. **(C)** Heat maps of spatial distribution of exploration over the 10-min three-chamber sociability test for each group (maps obtained from animals tested with the stimulus mouse placed in the left chamber of the apparatus). Warm colors (red/yellow) indicate areas of high occupancy; cooler colors (blue) represent zones less frequently visited. **(D,E)** Splash test performances. **(D)** Latency for the first grooming sequence. **(E)** Total grooming time (s). **(F)** Nest-building performance score. Inter-group comparisons were conducted with one-way ANOVA or Kruskal–Wallis tests, followed by Tukey’s or Dunn’s *post hoc* tests: *p < 0.05; ***p < 0.001; ****p < 0.0001; comparisons with a reference value 0 for the social index were conducted using a one sample t-test: $$$$ p < 0.0001; comparison between novel and familiar objects or mice were conducted using two-way ANOVA: #p < 0.05; ####p < 0.0001. Control n = 16; 3-hit n = 11; 3-hit ARZ n = 12.

Regarding the sociability test, the time spent interacting with each cage (Figure 4A) and the corresponding sociability index (Figure 4B) were assessed. During the habituation period (without stimulus), neither side nor group effects were observed for the time spent in the two side chambers (two-way ANOVA: side effect, F = 0.1614 p = 0.6891; group effect, F = 2.766 p = 0.0696; side × group interaction, p = 0.9656), indicating no baseline preference prior to the introduction of the social stimulus (data not shown). In contrast, analysis of the total exploratory time during the sociability test revealed a significant group effect (Kruskal–Wallis test, p = 0.0092). Post hoc Dunn’s tests indicated a significant reduction in exploratory time in the 3-hit group (p = 0.0460) and in the 3-hit ARZ group compared to controls (p = 0.0200). When the stimulus mouse was placed in one of the chambers, statistical analysis (two-way ANOVA) highlighted a side effect (F = 135.3 p < 0.0001), a group effect (F = 4.594 p = 0.0167), as well as an interaction between these two factors (F = 19.27 p < 0.0001) on the exploratory behavior. Control mice showed a significant preference for the social stimulus, spending significantly more time interacting with the cage containing the unfamiliar mouse than with the empty cage (Bonferroni’s post-hoc p < 0.0001). The sociability index globally differed between groups (one-way ANOVA, F = 37.72, p < 0.0001). Post hoc analyses indicated a significantly lower sociability index in the 3-hit group compared to controls (Tukey’s, p < 0.0001), and a significantly higher index in the ARZ-treated 3-hit group compared to untreated 3-hit mice (Tukey’s, p < 0.0001). The sociability index of ARZ-treated 3-hit mice did not differ from that of controls. Moreover, the sociability index of control mice was significantly above 0 (one sample t-test; p < 0.0001), indicating a significant motivation for the exploration of the cage containing a mouse instead of an empty one. Interestingly, 3-hit mice failed to show a clear preference for the social versus non-social cage. The time spent interacting with both cages was statistically similar, reflecting blunted social engagement. However, their sociability index even if very low (0.12 ± 0.04), was significantly above chance level (one sample t-test; p < 0.0201), suggesting a residual, though weakened, social drive. The sociability index of 3-hit ARZ group was also significantly above 0 (one sample t-test; p < 0.0001). Therefore, ARZ treatment reestablishes a normal social behavior in 3-hit animals. The heat maps (Figure 4C), which represent the spatial distribution of exploration, confirm these results by a qualitative view. The spatial pattern indeed shows a strong and focused interest for the chamber containing the stimulus mouse in controls, while the 3-hit mice showed a more dispersed exploration, with a tendency to remain in the central compartment of the arena or the corners of the apparatus, and less time near the social stimulus. The ARZ-treated 3-hit group displayed a heat map profile closely resembling that of control mice, with an increased presence in the chamber containing the social stimulus.

The splash test assesses self-care behavior through grooming behavior after spraying a 10% sucrose solution onto the animal’s fur. Significant group differences were observed in grooming latency (Kruskal–Wallis; p < 0.0001) and grooming duration (Kruskal–Wallis; p = 0.0273) (Figures 4D,E). Control animals showed a short grooming latency (mean latency: 1.29 ± 0.73 s), whereas 3-hit mice were significantly longer to initiate grooming (Kruskal–Wallis; p < 0.0001), associated with a reduced grooming duration compared to controls (Kruskal–Wallis; p < 0.0264). ARZ-treated 3-hit mice did not show significant differences in either grooming latency or total grooming duration when compared to untreated 3-hit mice or control animals, indicating an intermediary selfcare behavior between control and 3-hit mice.

To further assess goal-directed motivation, the nest building test was performed but did not reveal significant differences between the 3 groups (Figure 4F). All groups engaged in nest building to a comparable degree, regardless of condition or treatment. This suggests that not all dimensions of motivation are equally affected in the 3-hit model, and that motivational deficits may be task- or context-dependent.

## 4 Discussion

We recently described the behavioral profile of a new 3-hit model in female mice (Mouffok et al., 2024). In the present study, the predictive validity has been met through the demonstration of a beneficial effect of chronic ARZ treatment on cognitive deficits and negative-like symptoms. Furthermore, we have completed the analysis of face validity by assessing several behavioral-like symptoms mimicking those encountered in schizophrenia in both male and female mice. We have moreover strengthened the translational aspect of the model by extending the characterization of cognitive deficits and negative-like symptoms. Regarding negative symptoms and in addition to sociability deficits, we have particularly highlighted apathy-like behavior, apathy being frequently reported in schizophrenic patients (Awada et al., 2026), but not often assessed in animal models.

Regarding the predictive validity assessed herein, our results show that chronic administration of ARZ for 9 weeks, and initiated 3 weeks before the start of the behavioral assessment, allows (even if partially in certain cases) to improve the positive-, negative-like symptoms and cognitive deficits displayed by the 3-hit mice. A first element of discussion concerning the pharmacological aspect of this study concerns the dose and route used and the blood concentration reached during the behavioral evaluation period. In our conditions of administration via drinking solution, the blood concentration of ARZ after 9 days of treatment at the dose of 1 mg/kg, is approximately 10 times lower than after oral gavage administration for same duration and same dose (Chofflet et al., 2025). Thus, it is reasonable to extrapolate that the mode of administration used here is equivalent to a classic chronic oral administration of 0.1 mg/kg of ARZ per day. This dosage corresponds to a human dosage of approximately 4.9 mg/day, based on human equivalent dose calculation based on body surface area (Nair and Jacob, 2016). This is a low dose considering that the dosing range in clinic is 10–30 mg/day as a 1-time dose (Bowles and Levin, 2003). A hypothesis, related to pharmacodynamics, could be the preferential activation of certain receptors targeted by ARZ at this low dose/concentration. Indeed, this effect at a low dose is comparable to that observed by Nagai et al. (2009), who demonstrated after subchronic PCP administration in mice, that repeated administration (p.o. once a day for 7 consecutive days) of doses of 0.03 and 0.1 mg/kg of ARZ prevent object recognition memory deficits. Furthermore, these authors (Nagai et al., 2009) demonstrated that these anti-amnesic effects were notably related to the activation of 5-HT1A receptors. Given the high affinity of ARZ for 5-HT1A receptors (Kikuchi et al., 2021; Shapiro et al., 2003), it can be argued that at the concentration used here, the effect related to the activation of these receptors is predominant. It is therefore conceivable that the effects of ARZ on cognitive- and negative-like symptoms, could be related, under our experimental conditions, to the activation of these 5-HT1A receptors. One proposed mechanism is that by activating somatodendritic 5-HT1A receptors, ARZ reduced serotonin release and subsequently increased dopamine release in the cortex, which may participate to its efficacy on negative and cognitive symptoms of schizophrenia (Assié et al., 2005; Bortolozzi et al., 2007; Millan, 2000). Increasing dopamine levels in the medial prefrontal cortex (mPFC) is considered critical for the ability of atypical antipsychotics to improve negative symptoms of schizophrenia (Carlsson et al., 2001). Interestingly, ARZ has been shown to increase extracellular levels of monoamines in the prefrontal cortex in freely moving C57BL/6J mice (Zocchi et al., 2005) at a low dose (0.3 mg/kg), but not at higher doses (3 and 30 mg/kg). Similar results have been reported in rats: Tanahashi et al. (2012) demonstrated that intraperitoneal injection of ARZ (0.5 mg/kg) increased dopamine release in the mPFC, whereas 10 mg/kg decreased this release. Considering the pharmacodynamic profile (receptor affinity) of ARZ, a mechanistic hypothesis involving D2 and 5-HT2A receptors cannot be ruled out (Naderi et al., 2025; Oyamada et al., 2015). Regarding D2 receptors, it seems that the behavioral effects of ARZ observed in our study could also be due to the partial activation of these receptors.

To our knowledge, the behavioral effects of chronic administration of ARZ have not been studied to date in the SRKO mouse model or in stress-related model involving maternal separation in mice. Regarding the subchronic PCP model, only one study (Nagai et al., 2009) used repeated administration of ARZ. Interestingly, Nagai et al. used, like us, low doses of the antipsychotic drug which counteracted the impairment in novel object recognition memory. This suggests that in our study, low occupancy rates of ARZ’s target receptors and/or preferential binding to receptors for which the antipsychotic has high affinity may underlie the benefits on deficits induced by glutamatergic hypofunction.

Our data revealed that ARZ is more efficient in improving cognitive deficits and negative-like symptoms than in positive-like symptoms. Possibly, in our experimental conditions of chronic exposure to a low dose, ARZ would have particular effects on brain mechanisms underlying cognitive deficits and negative symptoms. Furthermore, the selective effects on certain behavioral deficits should not be overemphasized. Indeed, in the open field test, ARZ had no effect on increased distance traveled, and only partially counteracted the increased rearing behavior in the 3-hit mice. This indicates a partial improvement of positive-like symptoms.

Collectively and regarding the characterization of its predictive validity, the 3-hit model response to other antipsychotics will require to be tested. For example, an absence of efficacy against negative and cognitive symptoms would be expected for a typical antipsychotic like haloperidol (Adem et al., 2019; Stark et al., 2019). Conversely, it is expected that amisulpiride and carizaprine could improve negative-like symptoms (Krause et al., 2018).

Regarding positive-like symptoms, the hyperlocomotor phenotype observed in the 3-hit mice is a well-established behavioral hallmark across schizophrenia-related rodent models (Winship et al., 2019). The results of the present study indicate that the 3-hit model is suitable for measuring positive-like symptoms in both male and female mice.

Negative symptoms such as social withdrawal, apathy, and reduced motivation represent some of the most debilitating aspects of schizophrenia, and remain challenging to model. Regarding the results of the sociability test, we confirmed the results obtained previously in female mice (Mouffok et al., 2024) and characterized the same phenotype in 3-hit male mice. Interestingly, previous results obtained in our laboratory showed that 2-hit mice (SRKO + maternal separation) did not display sociability deficits (Lahogue et al., 2025). The results obtained here with a 3-hit model therefore suggest that for both male and female mice, the addition of the 3rd factor (*i.e.*, subchronic PCP) is necessary to induced deficits in sociability, strengthening the construct validity of our 3-hit model. Reduced grooming time and increased latency before grooming in the splash test was also observed in 3-hit mice, indicating decreased self-care and apathy-like behavior. This important result even reinforces the face validity of our model. Indeed, the prevalence of apathy is approximately 50% in schizophrenic patients and constitutes the most frequent negative symptom in schizophrenia (Evensen et al., 2012; Faerden et al., 2010; Mulin et al., 2011). The splash test is often used to assess depressive-like behaviors in rodents but appears to be scarcely used in models of schizophrenia. Its use in the characterization of negative-like symptoms is however very useful, given that apathy is not only present in depression (Steffens et al., 2022) but also in other psychiatric pathologies, and in particular schizophrenia as specified above. Moreover, if apathy can occur in absence of depression (Evensen et al., 2012; Kring et al., 2013), most studies revealing that a considerable proportion of schizophrenic patients exhibit both apathy and depression (Kirkpatrick, 2014). Interestingly, the splash test has, to our knowledge, never been implemented in SRKO mice or after subchronic PCP treatment. Besides, several studies revealed that maternal separation leads to a decrease in grooming duration in this test (Birmann et al., 2025; Khoei et al., 2025), suggesting that the apathy characterized in our 3-hit model could be partly related to its construction, including an early episode of maternal separation.

Cognitive deficits are central to schizophrenia and highly predictive of functional outcome. Memory is the cognitive domain showing the most pronounced deficits, with working memory and episodic memory appearing to be primarily and most prominently affected (Aleman et al., 1999; Fatouros-Bergman et al., 2014; Weiss and Heckers, 2001). In this context, it is essential to assess these categories of memory in the 3-hit model. This was done for spatial working memory through spontaneous alternation behavior in Y-maze, and for recognition memory with novel object recognition memory and social recognition memory, measuring the factual component of episodic-like memory. Concerning spatial working memory, we observed in male and female mice, similar deficits as those previously obtained in female mice (Mouffok et al., 2024). We already observed these deficits in the 2-hit model (SRKO + maternal separation) previously mentioned (Lahogue et al., 2025). This indicates that the addition of the 3rd factor (subchronic PCP) did not worsen the memory impairment, possibly due to a floor effect. Concerning object recognition and social recognition performances, the 3-hit model also displayed deficits, which were however not present in male mice from the 2-hit model (Lahogue et al., 2025), suggesting, as already reported above for social behavior, that at least for male mice, the interaction of the 3 factors is necessary to obtain the phenotype including deficits in object recognition memory performances. Patients with schizophrenia experience social withdrawal and impaired social recognition memory (Harvey and Lepage, 2014). The social recognition test has not been implemented in the 2-hit model (SRKO + maternal separation), but deficits in social recognition memory have been reported in SRKO male and female mice (Aguilar et al., 2021). In this last study, it is important to notice that the authors did not observe a deficit in social interaction (absence of preference to investigate the cage containing the conspecific instead of the empty cage), but a deficit in the ability to remember and distinguish a familiar mouse from a new one, which relies on cognitive functions (social recognition). In our study, the 3-hit model displayed not only sociability but also social recognition deficits, suggesting that memory deficits may be at least partly underpinned by social troubles in the first phase of the test, altering the familiarity acquisition process. However, the recognition deficit is not only observed in the social recognition test but also in the object recognition test, which is in favor of a dysfunction of the memory processes in itself (acquisition and/or consolidation and/or recall), which therefore affects the social and the non-social recognition performances in the 3-hit mice.

Overall, compared with the corresponding 1-hit models, the 3-hit model described here displays a broader and more integrated symptomatic profile, with alterations in multiple domains such as social and cognitive functions (Supplementary Table S1). Notably, social behavior impairments seem to result from the interaction between the factors, rather than from any single factor alone, underscoring the non-linear and multifactorial nature of the resulting phenotype. Available experimental data indicates that social behavior is globally unaffected in the SRKO model (Aguilar et al., 2021; Lahogue et al., 2025), and comparable observations have been reported following several protocols of maternal separation (Rombaut et al., 2023; Shin and Lee, 2023). By contrast, the effects of chronic PCP exposure on social behavior are heterogeneous across studies, with conflicting results reported in the literature (Brigman et al., 2009; Dogra et al., 2024). Taken together, these data suggest that the emergence of social deficits in the 3-hit model reflects convergence and interactions of multiple vulnerabilities rather than the impact of any individual factor effect. Regarding the molecular and cellular modifications induced by the 3-hit combination, they remain to be studied. However, such modifications have been reported for each of the factors used for the construction of the model (Supplementary Table S2).

This study presents some limitations. First, given the small number of animals of each sex (n = 5–6), we were unable to analyze the inter-sex differences in the 3-hit model neither for the deficits induced, nor for the aripiprazole effects. It would be interesting to increase the number of animals of each sex to compare data to the inter-gender differences observed in clinical practice and the potential differences in pharmacological sensitivity (Leger and Neill, 2016). The data presented here gives several indications that the 3-hit model is relevant for schizophrenia research. However, its symptoms profile also includes dimensions that are not specific to schizophrenia and are observed in other psychiatric disorders, for instance depression. Importantly, even if social deficits were observed, the 3-hit model did not exhibit an anhedonia-like phenotype, as assessed by the sucrose preference test which was not significantly affected (Supplementary Figure S1). Additional behavioral assays such as the forced swim test or resident-intruder paradigm, could provide complementary information regarding affective or social–agonistic behavior relevant to depression or bipolar disorder. Moreover, the effects of antidepressants or mood stabilizers, such as lithium, could be interesting to assess and to compare them with current antipsychotic treatments would be useful to further clarify the specificity of this model.

To conclude concerning the face validity, beyond further characterizing the spectrum of behavioral changes induced by this combination of 3 factors (particularly those affecting attentional processes, sensory filtering, and executive functions), neuropathological markers will need to be investigated (for instance, decreased cortical parvalbumin + GABAergic neurons, cortex and hippocampus atrophy, ventricles enlargement, long-term potentiation deficit). Moreover, given the neurochemical changes associated with schizophrenia, it would be judicious to measure levels of dopamine, serotonin, GABA and glutamate and their metabolites in the cortex, nucleus accumbens, hippocampus.

In conclusion, this study confirmed, from a behavioral perspective, the face and predictive validities of the 3-hit model with robust and clinically relevant persistent cognitive deficits and negative-like symptoms. This model, based on a powerful construct validity, is a promising tool to assess novel therapeutic strategies.

## Data Availability

The raw data supporting the conclusions of this article will be made available by the authors, without undue reservation.
