# Supplementary material for: Rescue of cognitive and negative-like symptoms by chronic aripiprazole treatment in a 3-hit mouse model of neurodevelopmental disorder
Source: Front Pharmacol. 2026 Mar 16;17:1760743. doi: 10.3389/fphar.2026.1760743 (PMC13033763; doi:10.3389/fphar.2026.1760743)
Supplement: Supplementary file 1 [file DataSheet1.docx]

Supplementary Material

# Supplementary Materials and Methods

**Sucrose preference test**

Anhedonia-like behavior was assessed using the sucrose preference test (Bouet et al., 2021; Pozzi et al., 2014). Animals were first individually housed and then habituated to the two-bottle choice procedure by providing free access to two bottles containing tap water for 24 h. After measurement of water intake over this 24-h period, animals were given free access for 72 h to two bottles, one containing a 2% sucrose solution and the other containing tap water. Bottle positions were alternated every day to prevent side preference. Fluid intake was measured by weighing the bottles, and sucrose preference was calculated as follows: sucrose preference (%) = [sucrose intake / (sucrose intake + water intake)] × 100.

# Supplementary Figures and Tables

## Supplementary Figure


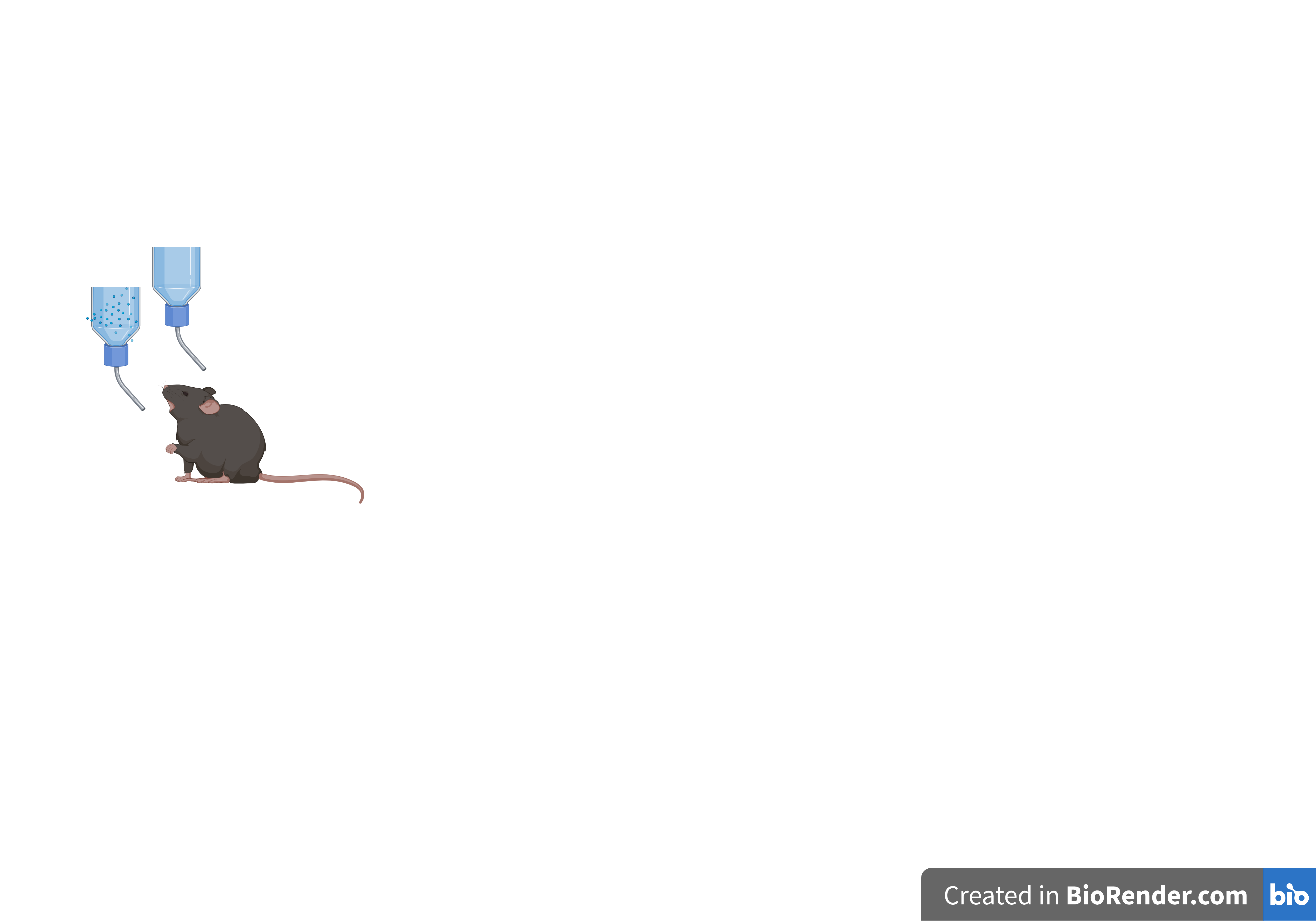


**Supplementary Figure 1. Sucrose preference test in the 3-hit model.**

Percentage sucrose preference was measured over 72 h. Data are shown as mean ± SEM with individual values overlaid. Comparisons with the 50% preference threshold were performed using a one-sample *t* test, $$$$ *p* < 0.0001. The control vs. 3-hit comparison using an unpaired *t*-test did not reveal any significant difference.

## Supplementary Tables

**Supplementary Table 1. Comparison of major behavioral alterations induced in SRKO or maternal separation or subchronic PCP and in 3-hit model in mice.**

| **Domain** | **Genetic vulnerability**  **SRKO** | **Environmental factor**  **Maternal separation** | **Pharmacological factor**  **Subchronic PCP** | **Multifactorial**  **3-hit model (SRKO + MS + PCP)** |
| --- | --- | --- | --- | --- |
| Locomotor behavior | Unchanged or mild effects | Inconsistent | Increased | Increased |
| Working memory | Impaired | Mildly impaired | Impaired | Impaired |
| Object recognition memory | Mostly preserved | Preserved or mildly affected | Impaired | Impaired |
| Social recognition memory | Impaired | Conflicting results | Impaired | Impaired |
| Sociability | Preserved | Preserved | Conflicting results | Decreased |
| Apathy / self-care | Not reported | Reduced grooming | Not reported | Reduced grooming and increased latency |
| Key articles | Aguilar 2021  Matveeva 2019  Lahogue 2025 | Bouet 2011  Fabricius 2008  Rombaut 2023  Shin 2023  Wu 2025 | Brigman, 2009  Dogra 2024  Gigg 2020  Noda 1995  Qiao 2001 | Mouffok 2024 |

**Supplementary Table 2. Referenced molecular and cellular changes in SR-KO, maternal separation, and subchronic PCP models**

| **Molecular/cellular alterations** | **Genetic vulnerability SRKO** | **Environmental factor Maternal separation** | **Pharmacological factor Subchronic PCP** | **Multifactorial  3-hit model (SRKO + MS + PCP)** |
| --- | --- | --- | --- | --- |
| Dendritic spines | Reduced | Reduced | Reduced | Not reported |
| BDNF | Reduced | Reduced | Reduced | Not reported |
| PV+ neurons | Conflicting results | Reduced during development | Reduced | Not reported |
| GABAergic synapses | Impaired | Reduced | Reduced | Not reported |
| PSD 95 | Not reported | Reduced | Reduced | Not reported |
| Key articles | Aguilar 2021 Balu 2011, 2013 Basu 2009 Benneyworth 2011 Jami 2021 Steullet 2017 | Irie 2024  Leussis 2012  Monroy 2010   Ohta 2020  Takatsuru 2009 Tan 2020 Tenkumo, 2020 Wang 2020 | Gigg 2020  Hajszan 2006 Katanuma 2014 Kjaerby 2014  Tanqueiro 2021 Zang 2016 | Not reported |

# References

Aguilar, D.D., Radzik, L.K., Schiffino, F.L., Folorunso, O.O., Zielinski, M.R., Coyle, J.T., Balu, D.T., McNally, J.M., 2021. Altered neural oscillations and behavior in a genetic mouse model of NMDA receptor hypofunction. Sci. Rep. 11, 9031.

Balu, D.T., Li, Y., Puhl, M.D., Benneyworth, M.A., Basu, A.C., Takagi, S., Bolshakov, V.Y., Coyle, J.T., 2013. Multiple risk pathways for schizophrenia converge in serine racemase knockout mice, a mouse model of NMDA receptor hypofunction. Proc Natl Acad Sci U S A. 110, E2400-9.

Balu, D.T., Coyle, J.T., 2011. Glutamate receptor composition of the post-synaptic density is altered in genetic mouse models of NMDA receptor hypo- and hyperfunction. Brain research, 1392, 1–7.

Basu, A.C., Tsai, G.E., Ma, C.L., Ehmsen, J.T., Mustafa, A.K., Han, L., Jiang, Z.I., Benneyworth, M.A., Froimowitz, M.P., Lange, N., Snyder, S.H., Bergeron, R., Coyle, J.T., 2009. Targeted disruption of serine racemase affects glutamatergic neurotransmission and behavior. Mol Psychiatry. 14, 719-27.

Benneyworth, M.A., Roseman, A.S., Basu, A.C., Coyle, J.T., 2011. Failure of NMDA receptor hypofunction to induce a pathological reduction in PV-positive GABAergic cell markers. Neurosci Lett. 488, 267-71

Bouet, V., Lecrux, B., Tran, G., Freret, T., 2011. Effect of pre- versus post-weaning environmental disturbances on social behaviour in mice. Neurosci. Lett. 488, 221–224.

Bouet, V., Percelay, S., Leroux, E., Diarra, B., Léger, M., Delcroix, N., Andrieux, A., Dollfus, S., Freret, T., Boulouard, M., 2021. A new 3-hit mouse model of schizophrenia built on genetic, early and late factors. *Schizophr. Res.* 228, 519–528.

Brigman, J.L., Ihne, J., Saksida, L.M., Bussey, T.J., Holmes, A., 2009. Effects of Subchronic Phencyclidine (PCP) Treatment on Social Behaviors, and Operant Discrimination and Reversal Learning in C57BL/6J Mice. Front Behav Neurosci. 3, 2.

Dogra, S., Aguayo, C., Xiang, Z., Putnam, J., Smith, J., Johnston, C., Foster, D.J., Lindsley, C.W., Niswender, C.M., Conn, P.J., 2024. Activation of Metabotropic Glutamate Receptor 3 Modulates Thalamo-accumbal Transmission and Rescues Schizophrenia-Like Physiological and Behavioral Deficits. Biol Psychiatry. 96, 230-242.

Fabricius, K., Wörtwein, G., Pakkenberg, B., 2008. The impact of maternal separation on adult mouse behaviour and on the total neuron number in the mouse hippocampus. Brain Struct Funct. 212, 403-16.

Gigg, J., McEwan, F., Smausz, R., Neill, J., Harte, MK., 2020. Synaptic biomarker reduction and impaired cognition in the sub-chronic PCP mouse model for schizophrenia. J Psychopharmacol. 34, 115-124.

Hajszan T, Leranth C, Roth RH., 2006. Subchronic phencyclidine treatment decreases the number of dendritic spine synapses in the rat prefrontal cortex. Biol Psychiatry. 60, 639-44.

Irie, K., Ohta, K.I., Ujihara, H., Araki, C., Honda, K., Suzuki, S., Warita, K., Otabi, H., Kumei, H., Nakamura, S., Koyano, K., Miki, T., Kusaka, T., 2024. An enriched environment ameliorates the reduction of parvalbumin-positive interneurons in the medial prefrontal cortex caused by maternal separation early in life. Front Neurosci. 17, 1308368.

Jami, S.A., Cameron, S., Wong, J.M., Daly, E.R., McAllister, A.K., Gray, J.A., 2021. Increased excitation-inhibition balance and loss of GABAergic synapses in the serine racemase knockout model of NMDA receptor hypofunction. J Neurophysiol. 126, 11-27.

Katanuma, Y., Numakawa, T., Adachi, N., Yamamoto, N., Ooshima, Y., Odaka, H., Inoue, T., Kunugi, H., 2014. Phencyclidine rapidly decreases neuronal mRNA of brain-derived neurotrophic factor. Synapse (New York, N.Y.) 68, 257–265.

Kjaerby, C., Broberg, B.V., Kristiansen, U., Dalby, N.O. 2014. Impaired GABAergic inhibition in the prefrontal cortex of early postnatal phencyclidine (PCP)-treated rats. Cerebral cortex. 24, 2522–2532.

Lahogue, C., Boulouard, M., Menager, F., Freret, T., Billard, J.-M., Bouet, V., 2025. A new 2-hit model combining serine racemase deletion and maternal separation displays behavioral and cognitive deficits associated with schizophrenia. Behav. Brain Res. 477, 115301.

Leussis, M.P., Freund, N., Brenhouse, H.C., Thompson, B.S., Andersen, S.L., 2012. Depressive-like behavior in adolescents after maternal separation: sex differences, controllability, and GABA. Dev Neurosci. 34, 210-7.

Matveeva, T.M., Pisansky, M.T., Young, A., Miller, R.F., Gewirtz, J.C., 2019. Sociality deficits in serine racemase knockout mice. Brain Behav. 9, e01383.

Monroy, E., Hernández-Torres, E., Flores, G., 2010. Maternal separation disrupts dendritic morphology of neurons in prefrontal cortex, hippocampus, and nucleus accumbens in male rat offspring. J Chem Neuroanat. 40, 93-101.

Mouffok, I., Lahogue, C., Cailly, T., Freret, T., Bouet, V., Boulouard, M., 2024. A New Three-Hit Mouse Model of Neurodevelopmental Disorder with Cognitive Impairments and Persistent Sociability Deficits. Brain Sci. 14, 1281.

Noda, Y., Yamada, K., Furukawa, H., Nabeshima, T., 1995. Enhancement of immobility in a forced swimming test by subacute or repeated treatment with phencyclidine: a new model of schizophrenia. Br J Pharmacol. 116, 2531-7.

Ohta, K.I., Suzuki, S., Warita, K., Sumitani, K., Tenkumo, C., Ozawa, T., Ujihara, H., Kusaka, T., Miki, T., 2020.The effects of early life stress on the excitatory/inhibitory balance of the medial prefrontal cortex. Behav Brain Res. 379, 112306.

Pozzi, L., Dorocic, I.P., Wang, X., Carlén, M., Meletis, K., 2014. Mice lacking NMDA receptors in parvalbumin neurons display normal depression-related behavior and response to antidepressant action of NMDAR antagonists. *PLoS ONE* 9, e83879.

Qiao, H., Noda, Y., Kamei, H., Nagai, T., Furukawa, H., Miura, H., Kayukawa, Y., Ohta, T., Nabeshima, T., 2001. Clozapine, but not haloperidol, reverses social behavior deficit in mice during withdrawal from chronic phencyclidine treatment. Neuroreport. 2001 Jan 22;12(1):11-5.

Rombaut, C., Roura-Martinez, D., Lepolard, C., Gascon, E., 2023. Brief and long maternal separation in C57Bl6J mice: behavioral consequences for the dam and the offspring. Front Behav Neurosci. 17,1269866.

Shin, S., Lee, S., 2023. The impact of environmental factors during maternal separation on the behaviors of adolescent C57BL/6 mice. Front Mol Neurosci. 16, 1147951.

Steullet, P., Cabungcal, J.H., Coyle, J., Didriksen, M., Gill, K., Grace, A.A., Hensch, T.K., LaMantia, A.S., Lindemann, L., Maynard, T.M., Meyer, U., Morishita, H., O'Donnell, P., Puhl, M., Cuenod, M., Do, K.Q., 2017. Oxidative stress-driven parvalbumin interneuron impairment as a common mechanism in models of schizophrenia. Mol Psychiatry. 22, 936-943.

Takatsuru, Y., Yoshitomo, M., Nemoto, T., Eto, K., Nabekura, J., 2009. Maternal separation decreases the stability of mushroom spines in adult mice somatosensory cortex. Brain research, 1294, 45–51.

Tan, Y, Fujita, Y, Qu, Y, Chang, L, Pu, Y, Wang, S, Wang, X, Hashimoto, K., 2020. Phencyclidine-induced cognitive deficits in mice are ameliorated by subsequent repeated intermittent administration of (R)-ketamine, but not (S)-ketamine: Role of BDNF-TrkB signaling. Pharmacol Biochem Behav. 188, 172839.

Tanqueiro, S. R., Mouro, F. M., Ferreira, C. B., Freitas, C. F., Fonseca-Gomes, J., Simões do Couto, F., Sebastião, A. M., Dawson, N., Diógenes, M. J., 2021. Sustained NMDA receptor hypofunction impairs brain-derived neurotropic factor signalling in the PFC, but not in the hippocampus, and disturbs PFC-dependent cognition in mice. Journal of psychopharmacology (Oxford, England), 35(6), 730–743.

Tenkumo, C., Ohta, K. I., Suzuki, S., Warita, K., Irie, K., Teradaya, S., Kusaka, T., Kanenishi, K., Hata, T., Miki, T., (2020). Repeated maternal separation causes transient reduction in BDNF expression in the medial prefrontal cortex during early brain development, affecting inhibitory neuron development. Heliyon, 6(8), e04781.

Wang, A., Zou, X., Wu, J., Ma, Q., Yuan, N., Ding, F., Li, X., Chen, J., 2020. Early-Life Stress Alters Synaptic Plasticity and mTOR Signaling: Correlation With Anxiety-Like and Cognition-Related Behavior. Frontiers in genetics, 11, 590068.

Wu, Z., Zhou, L., Fu, H., Xie, Y., Sun, L., Li, Y., Xiao, L., Zhang, L., Su, Y., Wang, G., 2025. Maternal separation during lactation affects recognition memory, emotional behaviors, hippocampus and gut microbiota composition in C57BL6J adolescent female mice. Behav Brain Res. 476, 115249.

Zhang, Q., Yu, Y., Huang, X.F., 2016. Olanzapine Prevents the PCP-induced Reduction in the Neurite Outgrowth of Prefrontal Cortical Neurons via NRG1. Scientific reports, 6, 19581.
